# Spatial autocorrelation signatures of ecological determinants on plant community characteristics in high Andean wetlands

**DOI:** 10.1038/s41598-022-18132-9

**Published:** 2022-08-12

**Authors:** Adriana Lozada, Angéline Bertin

**Affiliations:** Departamento de Biología, Facultad de Ciencias, Universidad de La Serena, Raúl Bitrán 1305, La Serena, Chile

**Keywords:** Community ecology, Wetlands ecology

## Abstract

Understanding how biological communities are shaped is a central tenet of community ecology. Recent evidence highlights the potential of decoupling diversity spatial autocorrelation into its positive and negative components to reveal community assembly processes that would otherwise remain undetected, as well as to improve understanding of their impacts on different facets of diversity. Yet, such approaches have only been implemented to investigate the effects of a few assembly drivers on a small number of diversity components. Here, we used high Andean wetland plant communities over a strong latitudinal gradient to investigate the effects of various ecological factors on spatial autocorrelation patterns of nine community metrics with different informative values, including measures of richness, dominance, evenness and beta-diversity. By combining Moran’s Eigenvector Maps, partial least squares structural equation modeling, and regression analyses, we revealed two groups of community parameters presenting contrasting spatial patterns due to specific sensitivities to ecological factors. While environmental variation and wetland connectivity increased positive spatial autocorrelation in richness and dominance-related parameters, species co-occurrence promoted negative spatial autocorrelation in evenness-related parameters. These results offer new insights regarding both how ecological processes affect species assembly, as well as the information captured by classical taxonomic parameters.

## Introduction

Understanding how biological communities are shaped is necessary for biodiversity conservation^1–3^, safeguarding ecosystem functions and services^4^, and predicting responses to global change^5^. A large component of current research in community ecology is thus devoted to disentangling processes involved in community assembly. However, gathering direct empirical evidence of the ecological processes at play is almost impossible under natural conditions^6^. As a result, they are usually assessed indirectly through pattern-process relationship approaches^7,8^, which rely on the premise that assembly mechanisms leave particular signatures on communities, and that ecologists are able to unequivocally identify these footprints.

Among the approaches for disentangling mechanisms underpinning community diversity^9–12^ that have gained popularity in recent years is the investigation of diversity patterns across multiple spatial scales. This strategy rests on the fact that assembly processes generate nonrandom spatial structures, leaving footprints at different spatial scales^12–15^. Two major groups of ecological processes can be distinguished in this respect: those with broad-scale consequences for species distribution and diversity and those with fine/local scale effects. The former group corresponds to processes generating positive spatial autocorrelations in community diversity, which refers to situations in which diversity levels at nearby locations are more similar than expected by chance^16^. Such processes include contagious processes^16,17^, such as dispersal, that generate influences from surrounding sites on community composition, and environmental filtering driven by broad-scale gradients, which similarly leads to commonalities among sites in close geographic proximity due to their high environmental similarity^16^. The latter group incorporates mechanisms such as biotic interactions with little or no effect beyond the local scale. Because such mechanisms can create singular patterns locally, they are assumed to drive dissimilarities among nearby sites, that is negative spatial autocorrelations^13^.

Recently, Biswas et al.^14^ proposed a conceptual framework that capitalizes on these characteristics to identify assembly processes operating at various spatial scales and analyze their spatial pattern effects on different diversity components. The approach relies on the decoupling of diversity spatial autocorrelation into its positive (S^+^(x)) and negative components (S^−^(x)). It has the potential to reveal impacts that would remain undetected without such partitioning. For instance, it revealed that environmental disturbance enhances negative spatial autocorrelation in community diversity^14,15,18^ without necessarily affecting overall spatial autocorrelation^15,18^. In addition, it showed that disturbance provokes stronger responses in evenness than in richness^14^. These examples demonstrate that the autocorrelation decomposition framework of Biswas et al.^14^ can both offer novel insights into how assembly processes affect community assembly and shed light on the ecological signals that different diversity indices are able to capture. Yet, it has so far only been applied to a few community characteristics and assembly drivers.

Here, we used the autocorrelation decomposition approach to characterize the impacts of a range of ecological factors on spatial patterns of taxonomic community parameters and identify which they alter the most. To do so, we analyzed plant communities from high Andean wetlands in the Norte Chico region in Chile, which offer an excellent model for the purpose of our study. Since these ecosystems are insular formations distributed across a marked aridity gradient, various processes are expected to leave imprints at a range of spatial scales. These include dispersal restriction and environmental filtering, two potentially important assembly mechanisms with predicted broad-scale imprints (Table 1), and ecological drift and plant-plant interactions with projected influences at the local scale (i.e., individual wetlands, Table 1). The effects of dispersal on S^+^(x) is suggested by the patchy and highly isolated nature of these habitats, which likely allows migration only between wetlands in relatively close geographic proximity, and may thus precipitate stronger similarities in community composition^19^ and diversity among closest sites^20^. The broad-scale impacts of environmental filtering are anticipated due to the pronounced latitudinal climate gradient in the region, which gradually transitions from Mediterranean in the south to arid in the north^21,22^. The importance of drift, the random changes in species relative abundances^8^, is substantiated by theory and empirical evidence suggesting that it is a major driver of diversity in island-type habitats^20,23,24^. Its actual importance may depend on landscape factors influencing population size within communities and thereby risks of demographic stochasticity^25^. As for plant-plant interactions, they are well established key drivers of alpine vegetation^26,27^. At high elevations, where assemblages are subjected to harsh climatic conditions, positive interactions in particular are expected to assume high importance^28^. They are promoted by facilitator organisms, such as cushion plants, that act as foundation species^29,30^, stabilizing and improving local abiotic conditions (e.g. soil nutrient content), supporting the growth of other plants, affecting species recruitment, and enhancing alpine plant diversity^29–32^.

**Table 1:** Expected spatial signatures of community assembly processes on different community characteristics.

Assembly process	Expected spatial signature	Effects on community characteristics	Most prominent expected pattern
Dispersal	**Driver of S**^**+**^**(x).** Dispersal is a contagious process, generating similarities among nearby communities	Dispersal is expected to increase alpha-diversity in general. The theory of Island Biogeography^54^ postulates a major effect of dispersal on richness. Analyses of plant, invertebrate and macroinvertebrate communities suggest a direct effect of migration on species richness^44^ Dispersal is also a main driver of community assembly increasing similarity among connected communities. Empirical studies indicate a major role of dispersal on community uniqueness^92–94^	Driver of S^+^(x) in richness and local contribution to beta-diversity
Environmental filtering	**Driver of S**^**+**^**(x) essentially.** Environmental filtering can provoke similarities among nearby communities through induced spatial dependence due to the non-random distribution of the environmental variables acting as filters. Such effects are expected to be important along environmental gradients **Occasional driver of S**^**−**^**(x)** When sharp environmental variations occur (i.e., as expected in high mountains), environmental filtering can drive dissimilarities among nearby communities	According to empirical evidence, environmental filtering more strongly influences species richness than evenness^14,41^ Environmental heterogeneity is also a common driver of community divergence^95–98^. A major role of environmental variation on community uniqueness is commonly reported^99–101^	Driver of S^+^(x) in richness and LCBD
Species interactions (competition / facilitation)	**Potential driver of S**^**−**^**(x).** Species interaction is a community assembly driver with no expected effects beyond the local scale. It can thus lead to dissimilarities among nearby communities	Both competition and facilitation alter species number and relative abundance^57^. However, theoretical and empirical evidence indicates that they have a more direct^44^ and stronger impact^14,102^ on evenness than on richness. Considering that competition is expected to alter dominance patterns in communities, strong effects can be expected on diversity metrics emphasizing dominance While species interactions can increase differences among ecological communities, their effects on ecological uniqueness are not established	Driver of S^−^(x) in evenness- and dominance-related indices
Ecological drift	**Driver of S-(x).** Process with no expected effects beyond the local scale. It can thus lead to dissimilarities among nearby communities	Ecological drift provokes random fluctuations in species abundances, lowers diversity within communities and increases differences among ecological communities^23^. The specific sensitivity of richness and abundance-related indices to drift is not well established	Driver of S^−^(x) in LCBD and alpha diversity parameters

S^+^(x): positive spatial autocorrelation, S^−^(x): negative spatial autocorrelation.

To investigate the spatial footprints of these processes on high Andean plant diversity, we analyzed how associated ecological factors (wetland connectivity for dispersal^33^, environmental variation for environmental filtering, wetland size for drift, and species co-occurrence for species interactions^23,25,34^ influence spatial patterns in community characteristics. Since assembly processes can alter different aspects of the communities (Table 1), we explored ecological factor effects on various alpha-diversity measures (see Supplementary Table S1 online), including measures of richness, dominance (Shannon entropy, Shannon diversity and Simpson diversity), evenness (Pielou’s evenness, Shannon evenness and Simpson evenness), as well as on a beta-diversity parameter (i.e., local contribution to beta-diversity, LCBD^35^). We postulate that ecological factors associated with broad-scale processes (wetland connectivity and environmental variation)^36^ will drive S^+^(x)^13,16^ and have stronger effects on richness-related parameters^37,38^ and LCBD (Table 1). In addition, we expect ecological factors associated with fine-scale processes (wetland size and species co-occurrence) to generate S^−^(x) in LCBD, evenness and dominance-related parameters^14,15^ (Table 1). To test these predictions, we analyzed positive and negative autocorrelation patterns of each of the community metrics using Moran Eigenvector Maps. Then, we used partial least squares structural equation modeling (PLS-SEM) to estimate the influence of the ecological factors (abiotic environmental variation, species co-occurrence, wetland connectivity, and size) on each plant community parameters. Finally, we investigated whether the influence of ecological factors drove negative or positive spatial autocorrelation in community parameters by investigating the effects of the PLS-SEM path coefficients after excluding potential confounding effects due to the relatedness of the community parameters.

## Results

### Spatial structure of the diversity parameters

**Figure 1:**
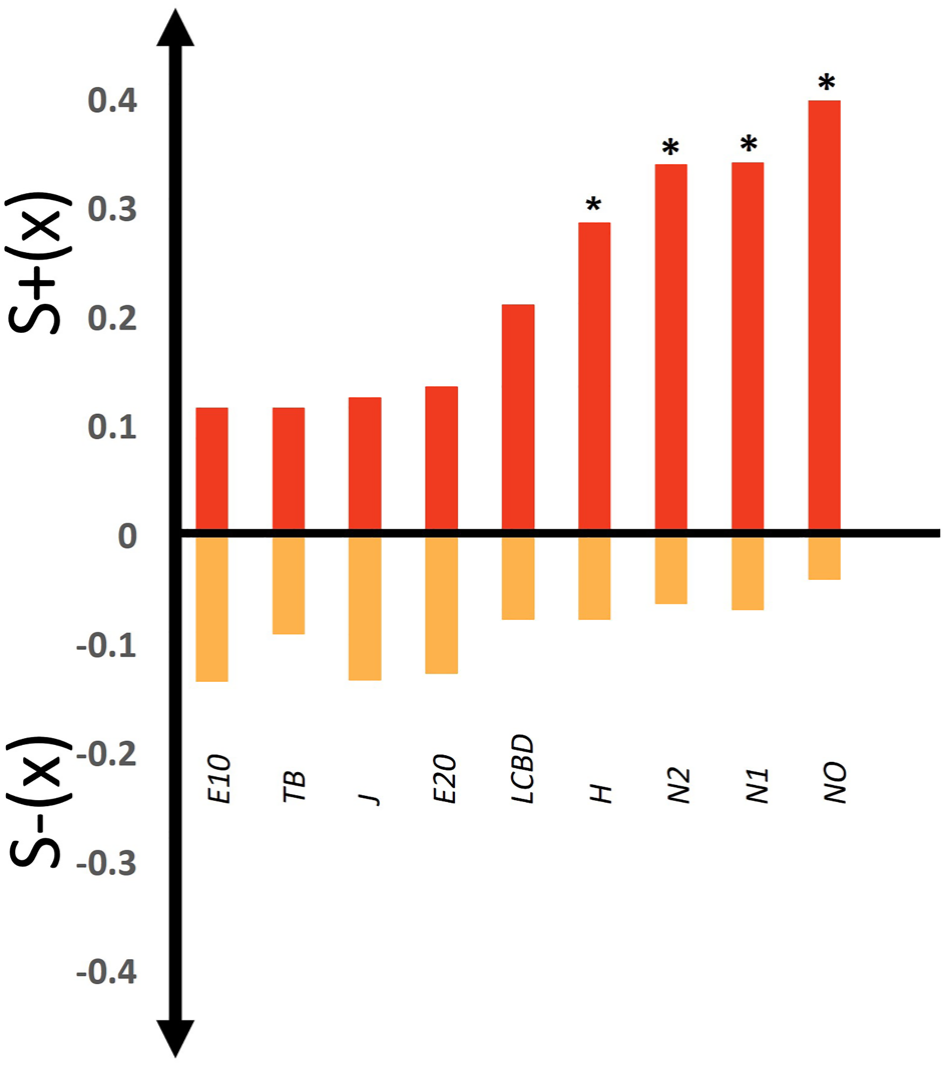
Levels of positive and negative spatial autocorrelation for the plant community parameters. Asterisks indicate significant autocorrelation (*P* < 0.05). *N0*: Richness; *H*: Shannon entropy; *N1*: Shannon diversity; *N2*: Simpson diversity; *E10*: Shannon evenness; *E20*: Simpson evenness; *J*: Pielou’s evenness, TB: Total biomass and LCBD: Local Contribution to Beta-Diversity.

All diversity indices displayed higher levels of S^+^(x) than S^−^(x) (Fig. 1). Substantial variation was revealed for both autocorrelation components, however, as indicated by coefficients of variation (CVs) of 50% and 38.5% for S^+^ and S^−^(x), respectively. Two groups of alpha-diversity indices stood out based on their spatial patterns. The first, comprising *N0*, *N1*, *H* and *N2,* displayed the highest and only significant levels of S^+^(x), but were characterized by the lowest levels of S^−^(x), while the second, comprising *E10*, *E20*, *J* and TB, demonstrated the opposite. Autocorrelation patterns of LCBD were intermediate between the two groups of alpha-diversity metrics. Overall, the community metrics displaying the highest S^+^(x) tended to be associated with low levels of S^−^(x), and vice versa, as indicated by the strong negative relationship between the two autocorrelation components (Pearson correlation: *r* =  − 0.90, likelihood ratio test of a mixed model of S^+^(x) in relation to S^−^(x) including the correlation matrix of community metrics as a random effect: *LR* = 16.62, *df* = 1, *P* < 0.001). Neither the levels of S^+^(x) nor those of S^−^(x) were influenced by the covariance of the community metrics (see Supplementary Table S2 online).

### Influence of local ecological factors on high Andean plant community characteristics

**Figure 2:**
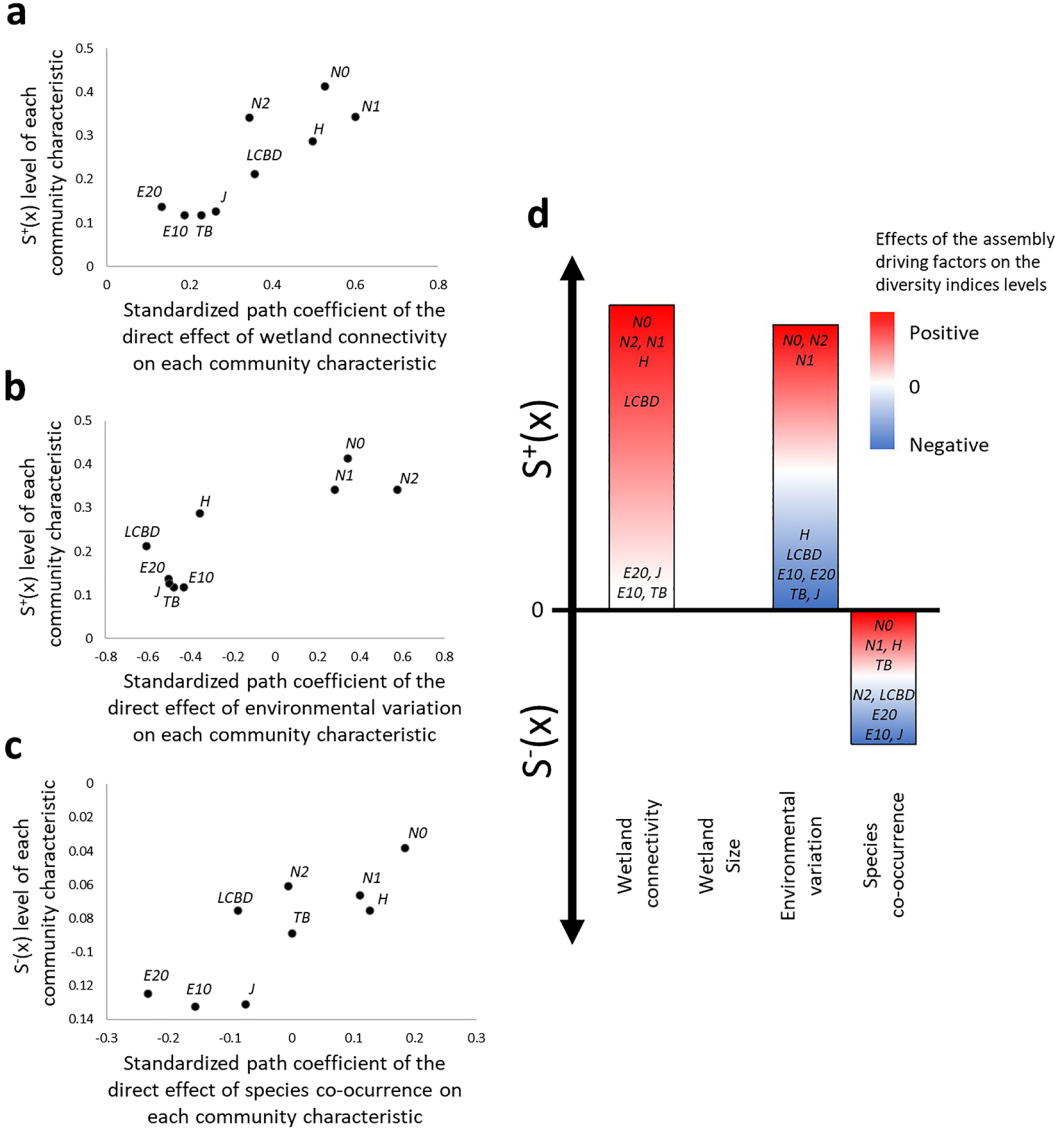
Relationships between the effects of ecological factors on community characteristics and the levels of spatial autocorrelation displayed by the latter. (**a**, **b, c**) Biplots for significant relationships of the autocorrelation levels of the community metrics by the amplitude of ecological factor’s path coefficients. Dots indicate the position of each community parameter. (**d**) Representation of the ecological factor effects. Bar sizes are proportional to detected effects. The colors indicate whether increases in autocorrelation are mediated by positive or negative effects of ecological factors on diversity indices. Positive effects are shown in red, negative in blue, and null in white. For instance, both wetland connectivity and environmental variation increased S^+^(x) of diversity indices that they positively influenced (i.e., *N0*, *N1*, *N2* and *H*). S^+^(x) and S^-^(x) refer to positive and negative spatial autocorrelations, respectively. *N0*: Richness; *H*: Shannon entropy; *N1*: Shannon diversity; *N2*: Simpson diversity; *E10*: Shannon evenness; *E20*: Simpson evenness; *J*: Pielou’s evenness, TB: Total biomass and LCBD: Local Contribution to Beta-Diversity.

Among the four ecological factors, only wetland connectivity and size had consistent effects on diversity indices (Table 2). Connectivity increased all community metrics, and significantly so in the case of *N1* and *N0*. In contrast, the estimated effects of wetland size were negative, indicating that increases in wetland size tended to produce a decrease in the community parameters and significantly so in *N0*. The impacts of environmental variation and species co-occurrence were inconsistent, with path coefficients ranging from negative to positive and characterized by very large CVs (i.e., 243 and 833% respectively, Table 2). Environmental effects were negative except for *N0*, *N1* and *N2* (Table 2, Fig. 2b). Species co-occurrence effects were positive for richness and dominance-influenced parameters (i.e., *H* and *N1*), but negative for evenness metrics (*E10* and *E20*). Overall, wetland connectivity and environmental variation had the strongest effects on the community parameters with mean absolute effects being at least three times higher than those of species co-occurrence and wetland size (e.g., mean of path coefficient absolute values in Table 2).

**Table 2:** Influence of wetland connectivity and size, species co-occurrence and environmental variability on each plant community parameter estimated by partial least squares structural equation modeling.

Index	Wetland connectivity		Wetland size		Species co-occurrence		Environmental variability	
	Standardized path coefficient ± SE	CI	Standardized path coefficient ± SE	CI	Standardized path coefficient ± SE	CI	Standardized path coefficient ± SE	CI
*N0*	**0.53** ± 0.32	[0.16, 1.50]	** − 0.23** ± 0.13	[**− **0.52, − 0.04]	0.18 ± 0.14	[**− **0.55, 0.0002]	0.34 ± 0.54	[**− **0.27, 1.53]
*H*	0.50 ± 0.34	[**− **0.06 1.53]	** − **0.13 ± 0.11	[**− **0.37, 0.08]	0.13 ± 0.24	[**− **0.58, 0.33]	** − **0.35 ± 0.53	[**− **1.53, 0.22]
*N1*	**0.60** ± 0.33	[0.12, 1.70]	** − **0.07 ± 0.11	[**− **0.32, 0.14]	0.11 ± 0.16	[**− **0.44, 0.19]	0.28 ± 0.50	[**− **0.29, 1.43]
*N2*	0.34 ± 0.32	[**− **0.32, 1.03]	** − **0.07 ± 0.10	[**− **0.26, 0.16]	** − **0.01 ± 0.16	[**− **0.29, 0.35]	**0.58** ± 0.66	[0.10, 2.14]
*E10*	0.19 ± 0.32	[**− **0.36, 0.93]	** − **0.02 ± 0.23	[**− **0.35, 0.52]	** − **0.16 ± 0.21	[**− **0.16, 0.67]	** − **0.43 ± 0.66	[**− **1.89, 0.23]
*E20*	0.13 ± 0.31	[**− **0.45, 0.81]	** − **0.06 ± 0.23	[**− **0.43, 0.47]	** − **0.23 ± 0.21	[**− **0.05, 0.77]	** − **0.50 ± 0.70	[**− **2.13, 0.08]
*J*	0.26 ± 0.31	[**− **0.30, 1.00]	** − **0.11 ± 0.19	[**− **0.48, 0.28]	** − **0.08 ± 0.25	[**− **0.33, 0.63]	** − **0.50 ± 0.59	[**− **1.89, 0.07]
TB	0.23 ± 0.36	[**− **0.20, 1.04]	** − **0.06 ± 0.15	[**− **0.38, 0.24]	0.001 ± 0.14	[**− **0.28, 0.32]	** − 0.48** ± 0.60	[**− **1.85, − 0.04]
LCBD	0.38 ± 0.28	[**− **0.04, 1.09]	0.02 ± 0.10	[**− **0.20, 0.19]	** − **0.09 ± 0.12	[**− **0.10, 0.39]	** − 0.61** ± 0.65	[**− **2.10, − 0.23]
Mean of path coeff. absolute values	0.35		0.09		0.11		0.45	
Path coefficient CV	46.68%		76.00%		832.69%		243.29%	

The confidence intervals and significance of path coefficients of each ecological factor were evaluated based on 10,000 bootstrapping iterations. Significant path coefficients are in bold. Models were carried out separately for each community metric. *N0*: Richness; *H*: Shannon entropy; *N1*: Shannon diversity; *N2*: Simpson diversity; *E10*: Shannon evenness; *E20*: Simpson evenness; *J*: Pielou’s evenness, TB: Total biomass and LCBD: Local contribution to beta-diversity.

### Effects of local ecological factors on positive and negative spatial autocorrelation components of diversity indices

Three of the four ecological factors increased spatial autocorrelation. Significant relationships between the ecological factor effects (i.e., PLS-SEM path coefficients) and levels of autocorrelation of the community parameters were actually revealed for wetland connectivity, environmental variation and species co-occurrence (Table 3). S^+^(x) levels showed a significant relationship with environmental variation and wetland connectivity effects, and the S^−^(x) levels with effects of species co-occurrence. Wetland connectivity and environmental variation both increased S^+^(x) of the community indices that they positively influenced (Table 3, Fig. 2a,b), and particularly *N0*, *N1*, *N2* and *H* (Fig. 2a,b,d). S^−^(x) levels increased in response to negative effects of species occurrence on community indices, leading to increased levels of S^-^(x) in *E10*, *E20* and *J* (Fig. 2a,c).

**Table 3:** Effects of ecological factors on autocorrelation levels of plant community parameters according to an exhaustive model selection approach based on the Akaike information criterion corrected for small sample size.

Spatial autocorrelation component	Adjusted *R*^*2*^	*F*	df_num,den_	*P*	Ecological factor	β ± sd	*t*	*P*
S^+^(x)	0.87	27.49	2.6	< 0.001	Wetland connectivity	0.064 ± 0.017	3.757	0.009
					Environmental variation	0.060 ± 0.017	3.566	0.012
S^−^(x)	0.63	14.34	1.7	0.006	Species co-occurrence	0.028 ± 0.007	3.786	0.007

Ecological factor effects were tested separately for the positive (S^+^(x)) and negative (S^−^(x)) spatial autocorrelation components of the community parameters. The models used the autocorrelation levels of each community parameter as the dependent variable and the PLS-SEM path coefficients measuring the direct effects of the ecological factors on each community characteristic as predictors.

## Discussion

By investigating the effects of ecological determinants on the spatial autocorrelation components of different diversity indices, we were able to reveal spatial footprints of assembly drivers and identify groups of diversity metrics presenting specific responses. These results thus provide new insights both on how ecological processes affect species assembly as well as the information captured by classical taxonomic diversity indices.

Here, we identified a range of drivers of S^+^(x) and S^−^(x) in high Andean plant communities. Environmental variation and wetland connectivity were two factors anticipated to promote similarity in community diversity among wetlands in close geographic proximity^19,39^. The fact that these factors had the strongest detected effects on community parameters is consistent with previous evidence that suggests they each play a key role in spatial patterns of high Andean wetland communities and diversity^19,20,39,40^. Their impacts on S^+^(x) in community metrics is also in line with literature associating dispersal restriction and environmental filtering with broad spatial scales^14,41^ when dispersal is a limiting factor in structuring communities^42^ and in the presence of environmental gradients^16^, as was the case here. Our results demonstrate that not all diversity components were equally influenced by these factors, however. Environmental variation and wetland connectivity increased similarity among wetlands in close geographic proximity in species richness and dominance-related indices (*N0*, *N2*, *N1* and *H*), but not in evenness. This adds to the mounting body of evidence showing that richness and evenness respond differently to a wide range of factors and mechanisms^14,37,41,43–50^. As predicted by theoretical literature and earlier empirical results^14,15,17,41^, we found that habitat connectivity increased species richness and S^+^(x) levels in this diversity component. High levels of connectivity also reduced community dominance (i.e., in *N1*), which is consistent with theoretical expectations that dominance increases with low migration rates, and *vice versa*^51^.

A noteworthy finding was that environmental effects promoted different spatial patterns on the community parameters depending on their specific impacts. They increased S^+^(x) in the community metrics that they positively influenced (i.e., richness and dominance-related parameters), which corroborates our expectations that environmental filtering acts at large spatial scale in our study system. However, and contrary to this expectation, no spatial signatures of environmental effect were revealed on biomass and LCBD, while both were significantly decreased by some abiotic variation. This highlights the existence of non-spatially structured physical variations of importance for the development and composition of high Andean wetland plant communities. High Andean landscapes are highly topographically heterogeneous displaying abrupt changes in local edaphic and microclimatic conditions^52,53^, which may lack clear spatial patterns at the resolution and scale of our study. Slope and mean annual wind speed, both ecological variables used in this study, are often associated with large local fluctuations and could thus alter plant community characteristics without leaving a specific spatial footprint.

Species co-occurrence was the only factor promoting S^−^(x) in plant community characteristics. Contrary to our expectations, wetland size had no such effect. The negative relationship between wetland size and richness in fact strongly differs from the premise that larger habitats hold a larger number of species as a consequence of higher levels of habitat diversity and propensity to support species from the regional pool^54,55^. Since our results contrast strongly with these expectations, it is suggested that vegetation extent, used here to assess wetland size, is not an adequate proxy for wetland capacity to support different species. The negative effect on richness instead suggests that it reflects the development of competitively superior species. Regarding the effects of species co-occurrence on S^-^(x), they were found to promote dissimilarities among geographically close wetlands in evenness indices (*E20*, *J* and *E10*). While species co-occurrences do not provide unequivocal proof of biotic interactions^56^, the detected effects are consistent with empirical evidence that species interactions affect evenness^14,37,44,57^, generate fine-scale patterns in diversity^15,58^ and play an important role in alpine plant community assembly^29,59^.

A striking finding of our study is the clear separation of two groups of alpha-diversity parameters presenting contrasting spatial patterns due to their specific sensitivities to ecological factors. Since our analyses discarded any confounding effects of the non-independency of the indices, this result cannot be considered as a simple mathematical artefact of their relationships, and thus demonstrates that assembly mechanisms tend to influence specific aspects of diversity. Understanding the informative value of diversity metrics is a longstanding issue in ecology^60–67^, and debate is ongoing regarding which measures are optimal^65,67–69^, as well as whether a single index could be universally applied for comparative purposes^69,70^. The fact that two groups of diversity metrics bore different spatial footprints demonstrates that each group is sensitive to different assembly processes, and encourages their combined use to aid in a better and more thorough understanding of the ecological processes that sustain community assembly. In addition, it shows that each diversity index holds specific potential for conservation and restoration purposes by motivating for either local or landscape-scale actions. While emerging trends from this and previous studies suggest that evenness tends to be particularly sensitive to local/fine-scale processes and richness to broader scale mechanisms, our understanding of how assembly factors generate and alter spatial patterns of different diversity facets is still limited to a few studies^14,15,18,71^. More empirical evidence from a wider range of ecosystems, including non-patchy habitats, is therefore required to establish the generality of these patterns. This will be essential to determining the specific potential of diversity parameters to decipher processes shaping communities.

## Methods

### Data collection and production

#### Sampling and processing of high Andean wetland plant communities

We used plant data of Bertin et al.^20^ and Pfeiffer et al.^40^ collected in 21 high Andean wetlands along a 600 km latitudinal range (between 26°S and 32°S) in north-central Chile between altitudes of 2852 and 4307 m a.s.l. (See Fig. 2 in Bertin et al.^20^). High Andean wetlands are permanent features formed by groundwater upwelling and occur in the low Alpine and sub-Alpine belts of the Central Andes^72^, fed by glacial streams, snowmelt, and rainfall^72^. These insular features contrast markedly from the arid grassland matrix in which they are found^73^. In the Norte Chico region of Chile, the location of this study, they occur along a latitudinal climatic gradient that varies from hyper-arid in the north to Mediterranean in the south, with mean annual precipitation extremes ranging from 35 to 200 mm, respectively. They support a wide variety plants and animals, including a number of endemic and rare species^72,74^.

Plant assemblages were characterized for each wetland from five 30 × 30 cm vegetation quadrats^20^. The biomass (g/m^2^) of each species was estimated by summing dried biomass of all individuals present within a quadrat after complete drying of the plant samples at 70 °C. A total of 52 species belonging to 21 families were identified. The plant database can be found as Supplementary Table S3 online.

#### Community parameters

Nine community metrics (see Supplementary Table S1 online) were estimated after pooling data from the five vegetation quadrats. They included total biomass estimated as biomass (dry weight in gr/m2) of all species present in the community samples, richness, three dominance-related metrics (Shannon entropy, Shannon diversity; Simpson diversity) and three evenness-related metrics (Shannon evenness, Simpson evenness, Pielou’s evenness) and the local contribution to beta-diversity calculated from the Hellinger transformed community data using the function beta.div in the adespatial package^35^.

#### Ecological variables

We assessed characteristics likely linked to environmental filtering, dispersal rates, drift, and species interactions. For environmental filtering effects, we used a subset of landform and climate related variables derived from the original datasets used by Bertin et al.^19^ and Pfeiffer et al.^40^, and which can be found in Supplementary Table S5 online. Only non-redundant variables with apparent influence on the diversity parameters were considered. These were identified by performing partial least square (PLS) regressions on the different diversity metrics. Only environmental variables that contributed strongly to the retained PLS components (defined using a leave-one out cross-validation procedure and by identifying the first local minimum of the root-mean-squared error^75^) were then retained for subsequent analyses. They included altitude, slope, mean annual wind speed (MAWS), mean annual number of snow days (SnowNDays), mean productivity (NDVI), and productivity variation (δNDVI).

We used graph-based connectivity metrics as variables related to dispersal rates. These were quantified for each of the wetlands in this study using GRAPHAB 1.0^76^, by considering all other wetlands occurring within a 20 km radius from the focal wetland according to the wetland map generated by Bertin et al.^20^. The connectivity measures calculated were node degree (Dg, the number of patches close to the focal patch^77^), closeness centrality (CCe, the mean distance from the focal patch to all other patches of its component^78^), eccentricity (Ec, the maximum distance from the focal patch to another patch of its component^79^), and betweenness centrality (BC, the sum of the shortest paths through the focal patch^76,80^). We used wetland size and species co-occurrence as proxies for drift risk and plant species interactions, respectively. Wetland size was assessed based on remotely-sensed NDVI derived from LandSat 8 OLI satellite imagery (see Bertin et al.^20^). Species co-occurrences were analyzed by calculating an average C-score for each wetland from the five plots. The C-score metric measures the level of segregation of all species pairs, which increases as average co-occurrences between all of the species pairs in the matrix decrease^81^. Thus, to allow an intuitive interpretation, the effects that we report for species co-occurrence are the additive opposite of the C-score effects. The C-score values were calculated with the R-package EcoSimR^81^.

### Statistical analyses

#### Spatial structure analyses of the plant community parameters

We estimated spatial autocorrelation components of the community parameters using Moran’s Eigenvector Maps^58^ as implemented in the R packages spdep^82^ and adespatial^83^. This framework allows producing spatial predictors, the MEMs, which model autocorrelation patterns at different spatial scales. They are obtained by eigen-decomposition of a spatial weighting matrix coding for connections between sites (i.e. wetlands in this instance), which produces orthogonal eigenvectors maximizing the spatial autocorrelation computed by Moran’s *I*^84^. When associated with positive eigenvalues, the MEMs represent positive autocorrelation and *vice versa*^13^. The spatial weighting matrix that we used to generate the MEMs considered connections among all wetlands for which strength was inversely proportional to their Euclidean distance. To estimate S^+^(x) and S^−^(x) of community indices, we followed the procedure described in Biswas et al.^14,15^, which involved summing the squared correlations between each diversity parameter and the positive and negative MEMs weighted by their associated eigenvalue.

#### Influence of ecological factors on plant community parameters

We investigated the influence of each ecological factor (wetland connectivity and size, species co-occurrence, and environmental variability) on community indices by carrying out PLS-SEM^85^. This is a comprehensive technique based on the use of a variable covariance matrix that identifies relationships and causality between variables^86^ with minimum requirements regarding measurement scales, sample sizes, and residual distributions^85^. The measurement model (i.e. the outer model, see Supplementary Figure S1 online) served to define latent variables representing the four ecological factors (wetland size, wetland connectivity, environmental variability, species co-occurrence) from the 12 measured ecological variables^87^. Environmental variability, wetland size, and connectivity were defined according to a formative mode and species co-occurrence using a reflective mode (see Supplementary Figure S1 online). Inspections of the loadings of the ecological variables on the latent variables indicated that the latent variables for wetland size and connectivity increased as wetland size and connectivity increased, respectively. The inner or structural model was constructed to estimate the effects of each of the four ecological factors on plant community characteristics (see Supplementary Figure S1 online). Eight separate PLS-SEM models were run to investigate these effects on each of the community metrics. Confidence intervals and significance of path coefficients were evaluated based on 10,000 bootstrapping iterations. These analyses were performed using the semPLS R-packages^85^.

#### Effects of assembly driving factors on positive and negative spatial autocorrelation components of community indices

To investigate whether the assembly driving factors influenced autocorrelation patterns of the community indices, we tested the relationship between the effects of the ecological factors (as estimated by their path coefficients) and the autocorrelation levels of the diversity parameters. To account for relatedness between the community parameters, we first analyzed the effects of their covariation on S^+^(x) and S^−^(x) levels. The pairwise Pearson’s correlation matrix of the plant indices was transformed through principal coordinate decomposition (PCoA^16,88^) using the ecodist R package^89^. Then, we analyzed the effects of the PCoA eigenvectors so-produced on S^+^(x) and S^−^(x) separately by carrying out generalized linear models using the R leaps package^90^. In these analyses, the autocorrelation levels of each community parameter were used as the dependent variables and the eigenvectors as the predictors. We ran the regsubset function, which performs an automatic selection procedure to look for the subset of eigenvectors that best explained the autocorrelation variation of the community indices. The best-fitting model, identified through a performance analysis performed with the AICcmodavg package^91^ and using the Akaike´s Information Criterion corrected for small sample size (AICc), was then tested for significance. Since these analyses did not reveal any impact of the relatedness between the community indices on their autocorrelation levels (see Supplementary Table S2 online), influences of the plant community parameters on S^+^(x) and S^−^(x) were analyzed through multiple linear regressions. The dependent variable of these analyses were the autocorrelation levels of each community parameter and the predictors the PLS-SEM path coefficients measuring the direct effects of the ecological factors on each community characteristic. For these analyses, we also performed an exhaustive model selection to search for the subset of ecological factors that best explained the autocorrelation levels using, as above, the regsubset function and based on the AICc criteria. Normality of the residuals was verified using Shapiro tests.

## Supplementary Information

Supplementary Information.

## Data Availability

All the data used in the analyses are provided in Supplementary Tables S3, S4 and S5 of the Supplementary Information file.
