# Supplementary material for: Spatial autocorrelation signatures of ecological determinants on plant community characteristics in high Andean wetlands
Source: Sci Rep. 2022 Aug 12;12:13770. doi: 10.1038/s41598-022-18132-9 (PMC9374769; doi:10.1038/s41598-022-18132-9)
Supplement: Supplementary file 1 — Supplementary Information. [file 41598_2022_18132_MOESM1_ESM.pdf]

Supplementary Information

Spatial autocorrelation signatures of ecological determinants on plant community characteristics in high Andean wetlands

Adriana Lozada and Angéline Bertin

Contents:

Figure S1

Table S1

Table S2

Table S3

Table S4

References

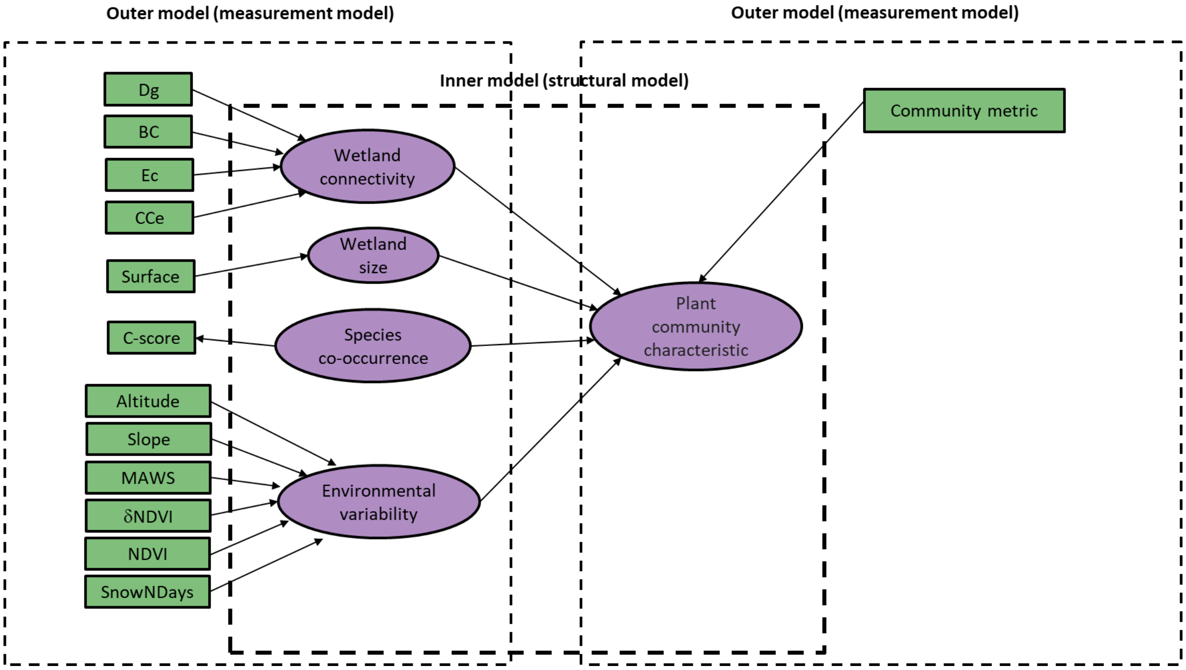

Figure S1. Diagrammatic representation of partial least squares structural equation model showing relationships and causality (direction of arrow) between latent and observed variables. The ovals identify latent variables and rectangles the measured variables. In total, nine models were run for each of the investigated community parameters separately (Richness, Shannon entropy, Shannon diversity, Simpson diversity, Shannon evenness, Simpson evenness, Pielou's evenness, total biomass and local contribution to beta-diversity). Dg: node degree; BC: betweenness centrality; Ec: eccentricity; CCe: closeness centrality; Surface: wetland surface; MAWS: mean annual wind speed; δNDVI: productivity variation; NDVI: mean productivity; SnowNDays: mean annual number of snow days.

Table S1. Community measures sed in this study and their description. S: number of species; p_i: relative abundance of the *i*th species, SS_{Total}: the total sum of square of species composition data and SS_j: sum of squares corresponding to the *j*th sampling unit. Codes are in accordance with Boccard et al.[1]

Community characteristic	Abbrev.	Formula	Description
Richness	*N0*	$N0 = \Sigma p_i^0 = S$	Total number of species in the community samples[2]
Shannon entropy	*H*	$H = - \Sigma [p_i * \log(p_i)]$	Equitability metric[3] measuring the uncertainty in species identity of a randomly chosen individual among the community samples[2]. A higher degree of uncertainty implies greater diversity[4]
Shannon diversity	*N1*	$N1 = \exp(H)$	Diversity metric that weights species according to their proportional abundances[5]. It can be interpreted as the effective number of species corresponding to *H* in the community samples[2].
Simpson diversity	*N2*	$N2 = 1/ \Sigma p_i^2$	Dominance metric that places more weight on the frequencies of abundant species and discounts rare species[6]. It increases as diversity increases[7].
Pielou's evenness	*J*	$J = H/\log(N0)$	Evenness metric measuring the relative abundance of species present in the community[1]. Increasing the uniformity of individual distribution among species leads to diversity increases.
Shannon evenness	*E10*	$E10 = N1/N0$	Evenness metric representing the Hill's ratio of Shannon evenness. It can be interpreted as the ratio of abundant species to all species[8].
Simpson evenness	*E20*	$E20 = N2/N0$	Evenness metric representing the Hill's ratio of Simpson evenness. It can be interpreted as the ratio of very abundant to abundant species[8].
Total biomass	TB		Biomass (dry weight in gr/m^2) of all species present in the community samples.
Local contribution to beta-diversity	LCBD	$LCBD_j = SS_j/SS_{Total}$	Partitioning of total beta-diversity into local contributions. It is a comparative indicator of ecological uniqueness of the sites[9]. High LCBD values indicate a unique community composition.

Table S2. Effects of the non-independency of the community parameters on their autocorrelation patterns. The pairwise Pearson's correlation matrix of the community metrics was transformed through principal coordinate decomposition, and the effects of the PCoA eigenvectors so-produced tested separately on $S^{+}(x)$ and $S^{-}(x)$. An automatic selection procedure to look for the subset of eigenvectors that best explained the autocorrelation variation of the community indices was performed (see main text).

	Model testing				Variables testing				
Dependent variable	Adjusted R2	*F*	df (num,den)	*P*	Selected variables	Estimate	Std. error	*t*	*P*
S+(x)	0.00	0.06	7,1	0.99	Eigenvector 6	-0.036	0.073	-0.494	0.708
					Eigenvector 5	0.033	0.117	0.287	0.822
					Eigenvector 4	0.040	0.180	0.220	0.862
					Eigenvector 8	0.024	0.278	0.085	0.946
					Eigenvector 3	-0.013	0.191	-0.066	0.958
					Eigenvector 1	0.007	0.196	0.034	0.979
					Eigenvector 2	-0.004	0.194	-0.020	0.987
S-(x)	0.00	0.06	7.1	0.99	Eigenvector 8	-0.034	0.081	-0.417	0.749
					Eigenvector 6	-0.007	0.021	-0.308	0.810
					Eigenvector 5	0.009	0.034	0.268	0.833
					Eigenvector 4	0.012	0.053	0.237	0.852
					Eigenvector 2	-0.003	0.057	-0.049	0.969
					Eigenvector 1	-0.001	0.057	-0.023	0.985
					Eigenvector 3	-0.001	0.056	-0.022	0.986

Table S3. Plant community data in the 21 high Andean wetlands of Chile´s Norte Chico. For each species and wetland, dried biomass (g/m2) was estimated from all individuals collected within the five quadrants. Wetland notation according to Bertin et al.[10]. The plant database can also be found at https://doi.org/10.5061/dryad.3p1tj06.

Plant species	S01	S02	S03	S04	S05	S06	S07	S08	S09	S10	S11	S12	S13	S14	S15	S16	S17	S18	S19	S20	S21
Acaena magellanica	0	0	0	0	0	1.74	0	0	0.31	0	0	6.55	0	0	0	0	0	0	0	0	0
Anagallis alternifolia	0	0	0	0	0	0	0	0.06	0	0	0	0	0	0	0	15.78	4.34	0.21	0.42	0	3.55
Arenaria rivularis	0	0	0	0	0	0	3.98	0.05	0	0	0.32	0	0	0	0	0	0.15	0.27	0.41	0	0
Astragalus bustillosii	0	0	0	0	0	0	0	0	0	0	0	0	0	0	0	0	2.6	0	10.22	6.31	0
Azorella trifoliolata	0	0	0	0	0	0	0	0	83.23	0	0	15.07	3.79	0	9.4	0	22.69	4.36	0.33	0	0
Baccharis juncea	1.84	0	0	0	0	0	0	0	0	0	0	0	0	0	0	0	0	0	0	0	0
Calandrinia compacta	0	0	0	6.9	0	0	0	0.89	0	0	0	0	0	0.02	0	0.01	0	0	0	0	0
Caltha sagitatta	0	0	0	0	0	0	0	0	0	0	0	0	23.11	0	0	0	0	0	0	0	0
Cardamine glacialis	0	0	0	0	0.06	0	4.69	0.01	0	0.24	0	0	0.04	0	0	0	0	0.72	0	0	0
Carex spp.	45.66	304.4	10.69	135	39.45	81.06	26.69	58.27	39.63	56.9	18.86	2.79	87.27	44.31	25.97	36.86	8.69	7.4	12.29	11.07	2.92
Carex vallis-pulchrae	0.55	0	17.23	0	0	0	0.04	13.3	6.58	0	5.43	6.62	1.34	138.4	0	0	0	0.13	25.58	146.3	4.58
Castilleja sp.	0	0	0	0	0	0	0	0	0	0	0	0	0	0.02	0	0	0	0	0	0	0
Colobanthus quitense	0	0	0	0	0	5.77	10.17	0	0	0	0	0	0	0	0.04	0	3.54	4.86	4.82	0	0
Deschampsia caespitosa	0	23.67	46.42	314.7	0	4.06	92.95	56.39	0	72.67	408.9	0	0	0	0	8.44	0	24.69	2.24	44.54	19.61
Deyeuxia velutina	0	103.4	230.8	1.36	0.12	21.16	14.41	0.74	10.22	6.49	0	0	27.14	166.5	34.71	0	0	0	15.82	159.4	140.2
Dicotyledonous sp.1	0	0	0	0	0.26	0	0	0.26	0	0	0	0	0	0	0	0	0	0	0	0	0
Distichlis spicata	0	0	0	0	0.02	0	0	0	0	0	0	0	0	0	0	0	0	0	0	0	0
Eleocharis palbibracteata	112.7	0	0	0	198.3	129.4	59.52	14.16	28.97	87	0	113.6	104.7	58.06	65.43	17.45	12.07	11.19	4.13	1.27	8.1
Eleocharis pachycarpa	25.23	0	0	0	0	0	0	0	0	0	0	0	20.56	13.74	0	0	0	0	0	0	0
Festuca kurtziana	0	0	0	0	0	0	0	0	0	0	0	0	0	0	0	0	0	0	0	0	246.1
Gastridium ventricosum	0	0	0	0	0	0	0	0	0	0	0	0	0	0	0	0	2.37	0	0	0	12.27
Gentiana sedifolia	0	0	0.55	0.31	0	0.01	2.43	2.16	4.71	0.8	0.04	0	0	0	0.13	0	0	0.25	0.89	0.59	0
Gramineae sp.	0	0	0	0	0	0	0	0	2.66	0	0	0	0	0	0	0	0	0	0	0	0
Hordeum sp.	13.56	50.69	0	0	18.19	4.58	0	0	0	1.64	0.79	0	0	1.96	0	9.04	11.49	0	0.04	0.73	0
Juncus articus	189.3	0	0	0	38.01	8.1	0	0	31.89	0.8	0	17.68	1.72	14.09	23.69	7.67	6.87	1.32	0	0	0
Juncus stipulatus	0	0	0	0	0	78.29	11.81	0	5.51	11.68	1.67	0.56	0	4.92	4.66	2.76	36.91	14.44	6.06	0.38	23.15
Lilaeopsis macloviana	38.48	0	17.48	0	0.42	0	0	0	32.79	0.21	0	59.27	0	0	0	4.08	2.67	0	0	0	0
Lobelia olygophylla	0	0	0	0	1.78	1.09	7.4	0	18.1	17.03	0	3.13	0.52	0.09	5.04	3.46	3.71	3.89	0	0.19	0
Mimulus luteus	0	0	0	0	0	0	0	0.1	0	0	0	0	0	0	0	0	0	0	0	0	0
Montia fontana	0	0	0	0	0	0	0	0	0	0	0	0	0	0	0.04	0	0	0.04	0.05	0.02	0.06
Muhlenbergia asperifolia	0	0	0	0	0.05	0	0	0	0	0	0	0	0	0	0	0	0	0	0	0	0
Moss	0	0	0	0	0	16.3	127.4	121.5	0	3.79	91.07	0	41.3	1.1	11.17	30.03	0	47.28	48.51	6.43	80.98

Oxychloe spp.	0	222.7	85	0	0	0	0	0	0	0	36.53	0	0	0	0	0	0	0	0	153.8	0.58
Patosia clandestina	0	0	0	0	0	0	1.98	9.2	175.7	256.8	0	0	0	127.3	0	0	0	0	112.9	8.66	154.4
Plantago barbata	0	5.56	0	0	0	0	2.69	2.25	0	0	0	0	0	0.24	3.54	0	4.59	9.82	34.8	19.55	6.59
Poaceae sp1	0	0	0	0	0	0.47	0	0	0	0	0	0	0	0.15	0	0	0	0	0	0	0
Poaceae sp2	0	0	0	0	0	0	0	0	0	0	0	0	0	0	2.48	0	0	0.43	0	0	0
Poaceae sp3	0	0	0	0	0	0.38	0	0	0	0	0	0	0	0	0	0	0	0	0	0	0
Poaceae sp4	0	0	0	0	0	0	0	0	0	0	0	0	0	0	0	13.96	0	0	0	0	0
Poaceae sp5	0	0	0	0	0	0	0	0	0	0	0	0	0	0	0	0	0	0	0	0	1.41
Poaceae sp6	0	0	0	0	0	0	0	0	0	0	0	0	0	0	1.6	0	0	0	0	0	0
Poaceae sp7	0	0	0	0	0	0.89	0	0	0	0	0	0	0	0	0	0	0	0	0	0	0
Poaceae sp8	0	0	0	0	0	0	0	0	0	0	0	0	0	0	0	0	0	0.58	0	0	0
Poaceae sp9	0	0	0	0	0	0	0	0	0	0	0	0	0	0	2.21	0	0	0	0	0	0
Poaceae sp10	0	0	0	0	0	0	0	0	0	0	0	0	0	0	4.55	0	0	0	0	0	0
Polygonum sp.	0	0	0	0	0	0	0	0	0	0	0	0	0	0	0	0	0	0	0	0	60.57
Puccinellia frigida	2.28	0.45	6.43	17.97	0.13	0.27	0.02	3.26	0.86	0.18	0.28	0	0	1.09	0.05	0.38	0.1	3.69	9.56	0.36	4.3
Ranunculus cymbalaria	7.18	5.78	0	0	18.18	0	0	0.26	0	0	0	0	0	0	0	0	0	0	0	0	0
Phylloscirpus acaulis	10.75	0	0	0	12.4	18.47	0.36	0	171.1	0	0	54.08	0	0.24	63.79	0	78.61	64.07	0	0	1.56
Trifolium repens	0	0	0	0	0	0	0	0	0	0	0	0	0	0.02	0.02	4.38	5.59	0	0	0	0
Triglochin palustre	0	64.33	29.27	0	0	0.18	0	0	0	0	0	0	0.07	0.09	0	0.38	3.79	0	1.41	0	0
Werneria pygmaea	0	0	0	0	0	1.05	0	0	4.53	3.66	0	2.65	0	0	0	0	84.69	8.23	81.94	1.48	68.66

Table S4. Wetland coordinates and values of the estimated community parameters. Parameter abbreviations as in Supplementary Table 1. Wetland notation according to Bertin et al.[10]. Plant richness as in Table S3 of Bertin et al.[10].

Wetland	Latitude (S)	Longitude (W)	*N0*	*H*	*N1*	*N2*	*E10*	*E20*	*J*	*TB*	*LCBD*
01	27° 03′	69° 20′	11	1.637	5.138	3.772	0.467	0.343	0.683	447.49	0.055
02	27° 05′	69° 10′	9	1.558	4.748	3.805	0.528	0.423	0.709	780.96	0.057
03	27° 04′	68° 55′	9	1.485	4.415	3.066	0.491	0.341	0.676	443.83	0.063
04	27° 29′	69° 05′	6	0.838	2.311	1.928	0.385	0.321	0.467	476.23	0.060
05	27° 47′	69° 29′	14	1.306	3.691	2.484	0.264	0.177	0.495	327.34	0.048
06	28° 01′	69° 33′	19	1.823	6.190	4.547	0.326	0.239	0.619	373.29	0.028
07	28° 36′	69° 57′	16	1.828	6.222	4.526	0.389	0.283	0.659	366.54	0.032
08	28° 38′	69° 54′	17	1.595	4.929	3.666	0.290	0.216	0.563	282.87	0.036
09	29° 28′	70° 04′	16	1.985	7.277	5.274	0.455	0.330	0.716	616.79	0.048
10	29° 36′	69° 59′	15	1.535	4.642	3.275	0.309	0.218	0.567	519.87	0.039
11	29° 50′	69° 55′	10	0.898	2.455	1.794	0.246	0.179	0.390	563.91	0.060
12	29° 59′	70° 02′	11	1.668	5.303	3.977	0.482	0.362	0.696	282.00	0.055
13	30° 23′	70° 24′	12	1.695	5.448	4.414	0.454	0.368	0.682	311.51	0.037
14	30° 33′	70° 17′	19	1.745	5.723	4.759	0.301	0.250	0.592	572.27	0.044
15	30° 49′	70° 21′	19	2.073	7.952	6.022	0.419	0.317	0.704	258.52	0.034
16	31° 00′	70° 19′	15	2.214	9.148	7.303	0.610	0.487	0.817	154.68	0.040
17	31° 16′	70° 33′	19	2.110	8.252	5.539	0.434	0.292	0.717	295.47	0.061
18	31° 27′	70° 34′	21	2.112	8.263	5.703	0.393	0.272	0.694	207.87	0.039
19	31° 32′	70° 33′	20	2.068	7.912	5.693	0.396	0.285	0.690	372.43	0.051
20	31° 36′	70° 36′	17	1.682	5.379	4.306	0.316	0.253	0.594	561.03	0.056
21	32° 14′	70° 21′	19	2.048	7.750	5.862	0.408	0.309	0.695	839.52	0.058

Table S5. Ecological variables used in the present study. C-scores and the graph-based connectivity metrics including node degree (Dg), betweenness centrality (BC), eccentricity (Ec), closeness centrality (CCe) were quantified as described in the main text. Mean annual precipitation (MAP, mm), mean average wind speed (MAWS, $m.s^{-1}$), number of days with snow cover (SnowNDays), mean annual temperature (MAT, ºC), soil moisture (TCI), slope, mean productivity (Normalized Difference Vegetation Index, NDVI) and productivity variation (δNDVI) were measured as described in Pfeiffer et al. The wetland surface estimates were calculated in Google Earth Engine Explorer based on Google Earth. Wetland notation according to Bertin et al.[10]. Altitude, MAP, MAWS, SnowNDays, MAT, TCI, Slope and Surface can also be found at https://doi.org/10.5061/dryad.3p1tj06.

Wetland	C-score	Dg	BC	Ec	CCe	Altitude	MAP	MAWS	SnowNDays	MAT	TCI	Slope	NDVI	δNDVI	Surface
S01	0.51	40	0.00	620915	408174	3024	38	3.57	2.6	80.42	2.76	4.73	33.65	0.42	26100
S02	1.17	72	174.25	620537	408724	3765	50	4.65	43.7	44.83	2.75	0.46	23.95	0.96	24300
S03	1.42	26	0.00	628877	417940	4307	62	4.96	48.5	25.75	4.56	1.49	26.45	0.97	37800
S04	1.93	47	0.03	579915	370086	4241	68	4.78	63	21.83	3.04	2.73	52.75	0.83	15300
S05	0.55	93	2960.96	539282	329382	2852	65	3.86	2.5	39.67	4.75	5.24	23.4	0.45	70200
S06	1.01	168	17.05	512028	304399	3697	65	5.07	37	42.75	0.64	6.88	26.05	0.45	13500
S07	0.47	251	1167.94	441902	247507	3752	79	5.86	59.2	23.83	2.64	5.12	20.15	0.46	18900
S08	1.08	233	364.78	437886	244341	3853	79	5.39	80.3	23.83	4.40	5.85	26.45	0.81	6300
S09	0.59	162	9.59	364205	182489	3287	122	4.48	46.2	-5.83	4.20	9.62	36.95	0.69	15300
S10	0.97	142	854.12	375906	174808	3692	138	3.47	56.4	-14.92	1.48	3.59	26.45	0.57	28800
S11	0.47	125	243.37	400177	163120	3959	155	3.62	111.4	-19.67	2.86	5.05	54.15	0.79	76500
S12	0.76	100	79.20	417289	154719	2948	104	3.27	33.5	32.33	2.16	11.92	40.6	0.35	29700
S13	0.64	441	447.34	468240	138161	3022	149	2.68	62.3	8.08	2.50	10.74	168.6	0.21	185400
S14	0.76	434	672.25	485198	131565	3166	164	5.02	66.6	-1.75	4.00	10.13	25.65	0.39	25200
S15	0.57	659	1721.10	513624	127049	3151	159	3.34	86.5	17.92	4.08	8.64	83.6	0.23	79200
S16	0.81	802	5755.80	532739	125946	3165	192	3.12	140.9	0.58	3.14	6.31	174.25	0.32	187200
S17	0.72	949	598.11	566996	132136	3083	200	3.65	123.6	19.75	1.69	5.94	29.25	0.5	52200
S18	0.53	805	2953.66	586462	137642	3272	220	3.18	140.7	14.17	3.36	6.63	220.15	0.75	196200
S19	0.7	719	8732.20	595583	141033	3482	239	3.66	160.5	10.33	2.36	5.48	75.1	0.81	86400
S20	1.04	612	15926.57	604151	145484	3406	239	3.46	118.1	10.33	2.68	10.9	34.4	0.43	18000
S21	1.03	851	1346.14	666754	185204	3170	298	2.75	141.2	16.00	4.04	7.63	50.2	0.25	62100

References

1. Borcard, D., Gillet, F. & Legendre, P. *Numerical ecology with R* (Springer International Publishing, 2018).
2. Chao, A., Chiu, C.-H. & Jost, L. Unifying species diversity, phylogenetic diversity, functional diversity, and related similarity and differentiation measures through Hill numbers. *Annu. Rev. Ecol. Evol. S.* **45**, 297-324 (2014).
3. Ingram, J. C. in *Encyclopedia of Ecology* 332-334 (Elsevier B.V., 2008).
4. Chao, A., Wang, Y. T. & Jost, L. Entropy and the species accumulation curve: a novel entropy estimator via discovery rates of new species. *Methods Ecol. Evol.* **4** (2013).
5. Pallmann, P. *et al.* Assessing group differences in biodiversity by simultaneously testing a user-defined selection of diversity indices. *Mol. Ecol. Resour.* **12**, 1068-1078 (2012).
6. Chao, A. *et al.* Rarefaction and extrapolation with Hill numbers: a framework for sampling and estimation in species diversity studies. *Ecol. Monogr.* **84**, 45-67 (2014).
7. Morris, E. K. *et al.* Choosing and using diversity indices: insights for ecological applications from the german biodiversity exploratories. *Ecol. Evol.* **4**, 3514-3524 (2014).
8. Molinari, J. A calibrated Index for the measurement of evenness. *Oikos* **56**, 319-326 (1989).
9. Legendre, P. & De Cáceres, M. Beta diversity as the variance of community data: dissimilarity coefficients and partitioning. *Ecol. Lett.* **16**, 951-963 (2013).
10. Bertin, A. *et al.* Genetic variation of loci potentially under selection confounds species-genetic diversity correlations in a fragmented habitat. *Mol. Ecol.* **26**, 431-443 (2017).
